# Effect of a multicomponent exercise programme (VIVIFRAIL) on functional capacity in frail community elders with cognitive decline: study protocol for a randomized multicentre control trial

**DOI:** 10.1186/s13063-019-3426-0

**Published:** 2019-06-17

**Authors:** Alvaro Casas-Herrero, Ivan Anton-Rodrigo, Fabricio Zambom-Ferraresi, Mikel L. Sáez de Asteasu, Nicolás Martinez-Velilla, Jaione Elexpuru-Estomba, Itxaso Marin-Epelde, Fernanda Ramon-Espinoza, Roberto Petidier-Torregrosa, Juan L. Sanchez-Sanchez, Berta Ibañez, Mikel Izquierdo

**Affiliations:** 1 Geriatric Department, Complejo Hospitalario de Navarra (CHN), Pamplona, Navarra Spain; 2 Navarrabiomed, IdiSNA, Navarra Institute for Health Research, Pamplona, Navarra Spain; 3 CIBER of Frailty and Healthy Aging (CIBERFES), Instituto de Salud Carlos III, Madrid, Spain; 4 Department of Geriatrics, Matia Fundazioa, San Sebastián, Gipuzkoa Spain; 5 Department of Health Sciences, Public University of Navarra, Av. De Barañain, s/n 31008 Pamplona, Navarra Spain; 6 Biodonostia Institute for Health Research, Grupo de Investigación en Atención Primaria, San Sebastián, Spain; 7 Geriatric Department, Hospital Universitario de Getafe, Getafe, Madrid Spain; 8 Navarrabiomed–CHN–UPNA, IdisNA, Red de Investigación en Servicios Sanitarios y Enfermedades Crónicas (REDISSEC), Pamplona, Navarra Spain

**Keywords:** Multicomponent exercise, Frail, Gait impairment, Functional capacity, Cognitive impairment

## Abstract

**Background:**

The benefit of physical exercise in ageing and particularly in frailty has been the aim of recent research. Moreover, physical activity in the elderly is associated with a decreased risk of mortality, of common chronic illnesses (i.e. cardiovascular disease or osteoarthritis) and of institutionalization as well as with a delay in functional decline. Additionally, very recent research has shown that, despite its limitations, physical exercise is associated with a reduced risk of dementia, Alzheimer disease or mild cognitive decline. Nevertheless, the effect of physical exercise as a systematic, structured and repetitive type of physical activity, in the reduction of risk of cognitive decline in the elderly, is not very clear. The purpose of this study aims to examine whether an innovative multicomponent exercise programme called VIVIFRAIL has benefits for functional and cognitive status among pre-frail/frail patients with mild cognitive impairment or dementia.

**Methods/design:**

This study is a multicentre randomized clinical trial to be conducted in the outpatient geriatrics clinics of three tertiary hospitals in Spain. Altogether, 240 patients aged 75 years or older being capable of and willing to provide informed consent, with a Barthel Index ≥ 60 and mild cognitive impairment or mild dementia, pre-frail or frail and having someone to help to supervise them when conducting the exercises will be randomly assigned to the intervention or control group. Participants randomly assigned to the usual care group will receive normal outpatient care, including physical rehabilitation when needed. The VIVIFRAIL multicomponent exercise intervention programme consists of resistance training, gait re-training and balance training, which appear to be the best strategy for improving gait, balance and strength, as well as reducing the rate of falls in older individuals and consequently maintaining their functional capacity during ageing.

The primary endpoint is the change in functional capacity, assessed with the Short Physical Performance Battery (1 point as clinically significant). Secondary endpoints are changes in cognitive and mood status, quality of life (EQ-5D), 6-m gait velocity and changes in gait parameters (i.e. gait velocity and gait variability) while performing a dual-task test (verbal and counting), handgrip, maximal strength and power of the lower limbs as well as Barthel Index of independence (5 points as clinically significant) at baseline and at the 1-month and 3-month follow-up.

**Discussion:**

Frailty and cognitive impairment are two very common geriatric syndromes in elderly patients and are frequently related and overlapped. Functional decline and disability are major adverse outcomes of these conditions. Exercise is a potential intervention for both syndromes. If our hypothesis is correct, the relevance of this project is that the results can contribute to understanding that an individualized multicomponent exercise programme (VIVIFRAIL) for frail elderly patients with cognitive impairment is more effective in reducing functional and cognitive impairment than conventional care. Moreover, our study may be able to show that an innovative individualized multicomponent exercise prescription for these high-risk populations is plausible, having at least similar therapeutic effects to other pharmacological and medical prescriptions.

**Trial registration:**

ClinicalTrials.gov, NCT03657940. Registered on 5 September 2018.

**Electronic supplementary material:**

The online version of this article (10.1186/s13063-019-3426-0) contains supplementary material, which is available to authorized users.

## Background

The progressive ageing of the population and the challenges surrounding the care of elderly people have become an emergency for all health systems. The aged population has resulted in an increase in the activity of health and social services, and the needs of this population are completely different compared to those of similar age groups 50 years ago. Nowadays, it is time for a clinical transition in health systems, moving from disease management to a more functional perspective, around the principles of integrated, coordinated, continued and patient-centred care [1]. In this context, the World Health Organization recently stated that health-related policies should be considered from the perspective of the elderly person’s functional capacity (intrinsic capacity) rather than the disease or comorbidity experienced at a single point in time [2].

**Fig. 1:**
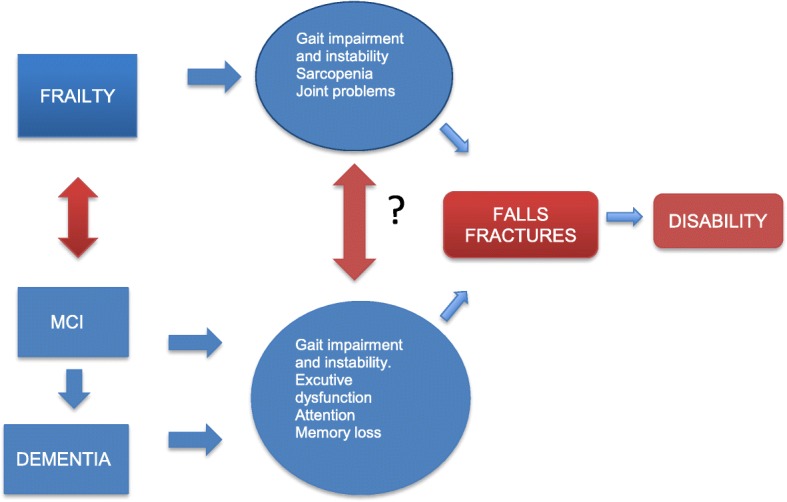
Proposed relation, sharing mechanisms and similar outcomes between frailty, mild cognitive impairment (MCI) and dementia

Frailty is a clinical syndrome defined by vulnerability and an increased risk of the individual to develop negative health-related events as disability and/or mortality under external stressors factors [3]. The frail phenotype pays attention to five domains (nutritional status, energy, physical activity, mobility and strength), and five criteria have been stablished (one per each domain: weight loss, exhaustion, leisure-time activity, gait speed and grip strength, respectively) [4] to identify older persons at high risk of numerous adverse outcomes [5, 6]. Some cross-sectional studies show the relation between frailty, mild cognitive impairment (MCI) and dementia, but the longitudinal relation is uncertain. Probably, the relation between cognitive impairment and frailty [7] in some cases is strong and bidirectional, since they share common pathophysiological bases and the same outcomes as hospitalization, falls, fractures, disability, institutionalization and mortality. Recently, the frailty Toledo study of aging has showed that cognitive impairment and muscular strength have a direct proportional relationship [8]. Therefore, dementia shares part of the same characteristics that can be found in the frail phenotype like subtle gait impairments (slow gait velocity and increase gait variability) and poor physical activity (Fig. 1).

The regular practice of physical exercise constitutes an inexpensive and healthy way of preventing and treating several illnesses related to the cardiovascular system and a sedentary lifestyle. Physical exercise programmes have been shown to be a novel “prescription tool” to prevent or delay the appearance of disability [9], to diminish healthcare costs [10] and to delay premature mortality [11, 12]. In the context of the ageing population, the performance of physical exercise can be considered the most effective intervention to delay disability and adverse events that are usually associated with frailty and vulnerable ageing. In some studies, exercise has shown even more positive results in terms of mortality from different diseases and chronic conditions than poly-pills for cardiovascular prevention [13]. Physical exercise, as an individual intervention, is one of the most important components in improving the functional capacity of frail seniors, and muscle strengthening in particular should be at the forefront of the treatment [14].

The benefit of physical exercise in ageing and particularly in frailty has been the aim of recent research. Moreover, physical activity in the elderly is associated with a decreased risk of mortality, of common chronic illnesses (i.e. cardiovascular disease or osteoarthritis) and of institutionalization as well as with a delay in functional decline. Additionally, very recent research has shown that, despite its limitations, physical exercise is associated with a reduced risk of dementia, Alzheimer disease or mild cognitive decline [15, 16]. Nevertheless, the effect of physical exercise as a systematic, structured and repetitive type of physical activity, in the reduction of risk of cognitive decline in the elderly, is not very clear. In this context, the most beneficial type of physical exercise is called multicomponent exercise [17–20]. This type of programme combines strength, resistance (aerobic), balance and flexibility training and has been shown to result in great improvements in functional capacity, which is a key point in maintaining independence in instrumental and basic activities of daily living.

Considering the relation between frailty and cognition previously mentioned (Fig. 1), it makes sense that those interventions proved to be effective in frail elderly people could be also effective in the elderly with cognitive impairment, and vice versa. Although there are still few studies, some of them show how resistance exercise programmes during 12 weeks in older adults not only induce improvements in gait velocity but also improvements in executive functions [21] which are directly related to fall risk [22]. Additionally, recent research [23] has shown that, despite its limitations, multicomponent exercise can be one of the best approaches to improving functional capacity and executive functions and to decreasing fall risk and cognition in frail institutionalized elderly with cognitive impairment and dementia [22]. Other authors have found similar results in terms of reversing the frailty status and cognitive improvements in community-dwelling elderly without basal cognitive impairment [24].

In this context, the purpose of this study aims to examine whether an innovative multicomponent exercise programme called VIVIFRAIL [25–27] has benefits for functional and cognitive status among pre-frail/frail patients with MCI or dementia. VIVIFRAIL was developed by world experts in the field of physical exercise and frailty, and is considered an important step towards the novel era of precise prescription of physical activity [26, 27]. Recently, it has been proven safe and effective to reverse the functional decline associated with acute hospitalization in very old patients [28]. This programme is a significant step towards an individualized physical exercise programme [27] according to the functional capacity of each elderly person and includes specific recommendations on doses (intensity, volume and frequency), similar to other treatments applied to vulnerable populations, such as frail elderly individuals with cognitive complaints.

## Methods

### Study design and setting

This study is a multicentre randomized clinical trial to be conducted in the outpatient geriatrics clinics of three tertiary hospitals in Spain. Geriatric clinics are part of routine geriatric clinical work and normally patients come from primary care or other specialities. Patients who meet the inclusion criteria will be randomly assigned to the intervention or control group. Prior to randomization, the attending geriatrician will review the absolute and relative contraindications to participate in the exercise programme and will provide general information about the study. Patient recruitment will begin with the normal visit of the patient to the clinic assessing for inclusion criteria and obtaining informed consent. Later on, subjects will be randomly assigned (as explained in the following) to either the intervention or the control group. The researcher who decides whether the patient is assigned to the intervention or control group will not be the attending geriatrician. Patients or their relatives (if the patient has dementia) will be informed of the random inclusion in one group but will not be informed regarding to which group they belong. The data for both the intervention group and the control group will be obtained at three different times: at baseline and at the 1-month and 3-month follow-up. We will perform two telephone calls (15 days and 2 months after baseline) to assess adherence and reinforce the protocol applied. In addition, in order to improve the adherence of the exercise programme, participants in the intervention group will record a diary that will be reviewed during follow-up. After randomization, the research team (physiotherapist, sport science specialist and geriatrician) will together perform the baseline measurement and follow-up visits of functional, pharmacological, comorbidity and cognitive assessment, as well as of mobility and strength evaluations (Table 1). The multidisciplinary research team have well-known previous experience in functional geriatric assessment and in the prescription of exercise in frail aged participants in different clinical settings [25, 26, 28].

**Table 1:** Schedule for the different primary and secondary variables for the participants of the study

Measure	Screening	T1 Baseline	T2 1 month	T3 3 months
Primary outcome				
Short Physical Performance Battery (SPPB)		X	X	X
Secondary outcomes				
Barthel Index	X		X	X
Frailty according to the Fried criteria	X			
Montreal Cognitive Assessment (MOCA)—only for mild cognitive impairment	X		X	X
Categorical scale of pain		X		X
Geriatric Depression Scale of Savage (GDS)		X		X
Minimental Cognitive Exam (MEC-Lobo)		X	X	X
Gait velocity test (GVT)		X	X	X
Dual task (verbal and counting GVT)		X	X	X
Maximal isometric force of handgrip, knee extension and hip flexion		X	X	X
Isaacs Set Test		X		X
Quality of Life (EQ-5D)		X		X
Trail Making Test (TMT—Part A)		X	X	X
1RM (leg press)		X	X	X
Muscle power at 50% 1RM in leg press		X	X	X
Drugs, geriatric syndromes and psycho-behavioural symptoms		X		X
Cumulative Illness Rating Scale for Geriatrics (CIRS-G)		X		
Functional Activities Questionnaire (FAQ)		X	X	X
Mini Nutritional Assessment (MNA)		X		
Acceleration data: gait kinematic parameters (regularity, variability, cadence), Five Times Sit to Stand Test (peak power, impulse) and balance parameters (power spectrum, area)		X	X	X
Rate and risk of falls		X	X	X
Mortality		X	X	X
Admission and readmission to hospital		X	X	X
Institutionalization		X	X	X

**Fig. 2:**
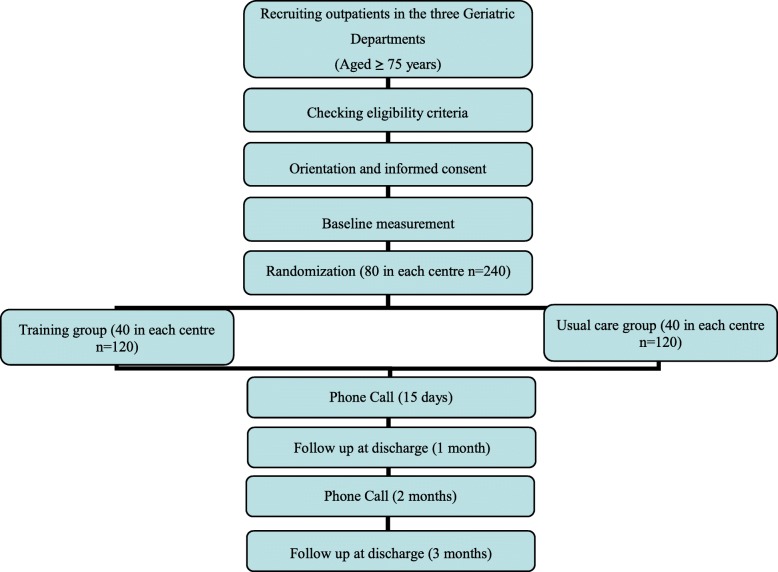
Flow diagram of the study protocol

Basic sociodemographic data of the participants will be collected in the baseline visit. All adverse events, including those related to exercise such as muscle pain, fatigue and general aches and pains, will be recorded in an “adverse events diary” during follow-up visits and telephone calls by the training and testing staff, and by self-report during the study period. The study flow diagram is shown in Fig. 2. The time at which different variables (primary and secondary) will be measured is presented in Table 1. The protocol employs relevant standard protocol items for clinical trials according to the SPIRIT 2013 statement (Additional file 1) [29] and follows the CONSORT statement [30] for transparent reporting. The trial is registered at ClinicalTrials.gov (identifier number iNCT03657940), and the status is recruitment.

### Study participants and eligibility criteria

The study will include outpatients of the Geriatrics Department of the Complejo Hospitalario of Navarra, the Matia Foundazioa in San Sebastian and the Hospital of Getafe (all three in Spain) older than 75 years of age between September 2017 and May 2019. Inclusion criteria include patients aged 75 years or older being capable and willing to provide informed consent, able to communicate and ambulate with or without personal/technical assistance, with a Barthel Index ≥ 60 and MCI or mild dementia according to DSM V criteria [31], level GDS-4 (Reisberg classification) [32], pre-frail or frail according to the Fried criteria [4] and having someone to help to supervise them when conducting the exercises.

The exclusion criteria include having any factor that precludes the performance of the physical training programme or testing procedures as determined by the attending physician. These factors, according to the VIVIFRAIL programme [26] include, but are not limited to, the following:

- myocardial infarction in the past 3 months;
- unstable angina pectoris;
- terminal illness;
- uncontrolled arrhythmia;
- unstable cardiovascular disease or other unstable medical condition;
- uncontrolled arterial hypertension;
- recent pulmonary thromboembolism;
- upper or lower extremity fracture in the past 3 months;
- institutionalized or pending entry into institution; and
- unwillingness to either complete the study requirements or to be randomized into the control or the intervention group.

### Randomization and blinding

Participants in the study will be randomized (www.randomizer.org) into an intervention group and a usual care group (control group) following a simple randomization procedure, in a 1:1 ratio without restriction. Participants will be explicitly informed and reminded not to discuss their randomization assignment with the assessment staff. The assessment staff will be blinded to the participants’ groups as well as to the main study design and to what changes in study outcomes we expect to occur in either group.

It will not be possible to conceal the group assignment from the staff involved in the training of the intervention group. Patients (or their families) will be informed of their random inclusion in one group but will not be informed regarding to which group they belong.

### Statistics and sample size

Assuming an alpha error of α = 5%, a correlation between pre and post-intervention values of the Short Physical Performance Battery (SPPB) of ρ = 0.5 and a standard deviation for the SPPB of σ = 2.5, the required sample size to have a power of 90% to detect a minimum difference of 1 point between groups in the post–pre SPPB score is 101 patients per group. Taking into account an expected loss of patients along the follow-up of 15%, the final sample size required is 120 per group. For the estimation, an ANCOVA method for the analysis of the differences has been considered. The research team will be focused to instruct and remind geriatricians periodically to assess inclusion and exclusion criteria in all three centres in order to reach target sample size. All of the centres have a remarkable clinical activity attending around 1000 patients every year. It is expected than 15% of these patients in every centre can be potentially eligible participants. In the unlikely case of not reaching the expected sample size, we will extend the inclusion period for a further 3 months.

The description of the sample by group will be conducted using statistics such as means and standard deviations or medians and interquartile ranges for the quantitative variables, and frequencies and percentages for the qualitative variables. For comparisons between groups at baseline, *t* tests or Mann–Whitney *U* tests will be used for continuous variables, depending on normality, which will be checked for each using the Kolmogorov–Smirnov test and normal probability plots, and the chi-square test or Fisher’s test will be used for categorical variables. To determine the efficacy of the intervention in the quantitative variables, such as the SPPB, we will use ANCOVA models, using post-intervention values as dependent variables, group study as the principal effect and pre-intervention values as covariates. In the case of qualitative or categorized variables (such as whether an improvement of a given magnitude between pre and post intervention has been achieved or not), comparisons between groups will be conducted with the chi-square test or Fisher’s test, and complemented with logistic regression if additional adjustment is needed. The level of statistical significance will be 0.05. Data will be analysed using an intention-to-treat approach with R and SPSS statistical packages.

We will adopt a complete case analysis as the primary analysis if the proportions of missing data are equal to or below 5% and it is implausible that certain patient groups specifically are lost to follow-up in one of the compared groups. To check the assumption that the potential impact of missing data is negligible (created by a missing complete at random or a missing not at random mechanism), best–worst and worst–best case sensitivity analyses will be used. When the proportions of missing data are above 5% and if this is supported by the previous sensitivity analysis, a missing at random mechanism will be assumed and missing values will be imputed using the R package MICE (Multivariate Imputation via Chained Equations). If the proportions of missing data are very large (more than 40%) on important variables, then trial results will be considered as hypothesis-generating results.

### Data management

Completed personal data or other documents containing protected personal health information will be kept in a locked file at the principal investigator office in every centre. Data will be entered into an electronic de-identified database by authorized study team members, and checked for completeness and accuracy. Access to data with identifiers will be restricted to authorized study team members and authorities. Electronic data will be stored on a secure server regulated by the local research institute (IDISNA). Identifiable data will be destroyed 10 years after study finalization or 5 years after last publication.

### Detailed description of the intervention

Participants will be randomly assigned to the following groups.

#### Usual care group (control)

Participants randomly assigned to the usual care group will receive normal outpatient care, including physical rehabilitation when needed.

#### Multicomponent VIVIFRAL exercise group

The multicomponent physical exercise programme will be the VIVIFRAIL programme which was developed in Europe (Erasmus+ programme of the European Union). The VIVIFRAIL [26, 27] multicomponent exercise intervention programme consists of resistance training, gait re-training and balance training, which appear to be the best strategy for improving gait, balance and strength, as well as reducing the rate of falls in older individuals and consequently maintaining their functional capacity during ageing [26, 27]. This type of intervention has also been proven as the most effective to delay disability, cognitive impairment and depression [33] as well as effective to reverse the functional decline associated with acute hospitalization in very old patients [28].

The VIVIFRAIL exercise programme offers a general guideline to design a multicomponent physical exercise programme for frailty and falls treatment and prevention among people aged older than 70 years [26]. The programme is personalized, depending on the older person’s functional capacity level (serious limitation, moderate limitation and slight limitation as evaluated by the SPPB and a walking speed test) and the risk of falling. VIVIFRAIL works on the following components of physical fitness: arm and leg strength and power, balance and coordination to prevent falls, flexibility and cardiovascular endurance (i.e. walking).

All of the exercises outline the procedure, guidelines for starting, frequency and progression to be able to correctly monitor the instructions prescribed to the patient and improve their health [26].

**Fig. 3:**
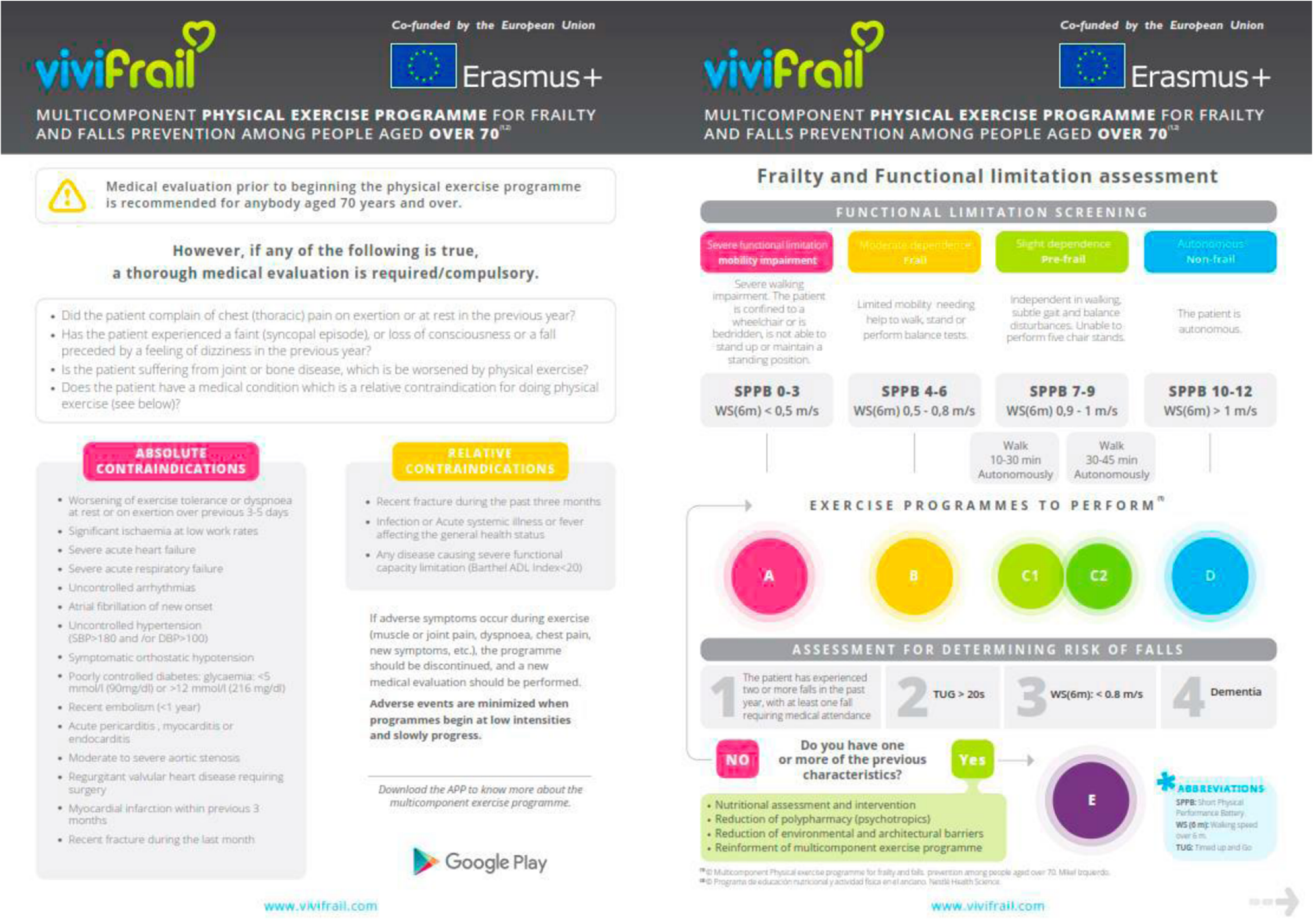
Classification by allocation of the participant to a physical exercise group by the SPPB, gait speed and risk of falls. Modified from Izquierdo et al. [26]

To individualize the exercise programme we will assess the initial functional capacity and determine the risk of falling. Different functional capacity levels will be determined based on the scores obtained from the Short Physical Performance Battery Test (SPPB) and the 6-m gait velocity test (GVT) (see Fig. 3), with each leading to the recommendation of a certain customized multi-component physical exercise programme (Program A, B, C1, C2 or D) (see Fig. 3). Two sub-types will be defined in order to more accurately recommend a particular cardiovascular endurance programme for the group with slight limitation (frail/pre-frail) based on the maximum time they can walk without help. If the person can walk for 10–30 min, they are known as C1; and if they can walk for 30–45 min, they are known as C2 [26].

This classification will be complemented according to the risk of falling, assessed by the answer to some simple questions (see Fig. 3). If a risk for falling is detected, the frequency of the exercise will be increased and some additional measures will be adopted, as depicted in the lower part of the right-hand side of Fig. 3. Finally, the participant will be assessed for potential contraindications or need of medical evaluation (left-hand side of Fig. 3) [26].

**Fig. 4:**
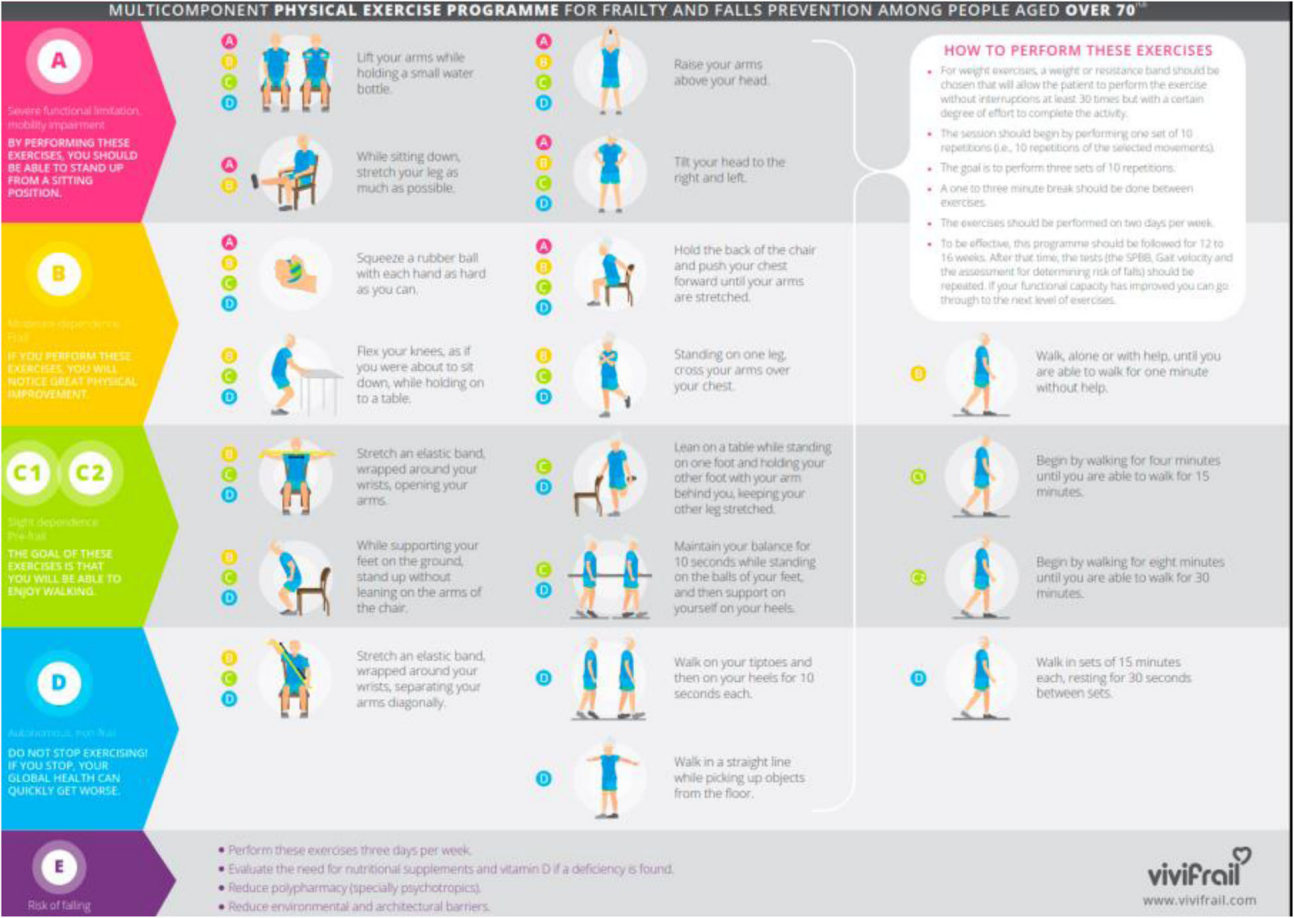
Classification by allocation and examples of exercises of the participant according to a functional level group by the SPPB, gait speed and risk of falls. Modified from Izquierdo et al. [26]

Once the patient has been assigned to one of the six categories, a multicomponent programme taking 12 weeks will be implemented. The general characteristics of the different programmes are shown in Fig. 4.

##### Resistance training

- Sets and repetitions:
  - ◦ The session should begin by performing two sets of 10 repetitions (i.e. 10 repetitions of the selected movements).
  - ◦ The goal is to perform three sets of 12 repetitions.
  - ◦ A 1-min to 3-min break should be taken between exercises.
  - ◦ The exercises should be performed on 3 days per week.
- Intensity and progression:
  - ◦ For weight exercises, a weight or resistance band should be chosen that will allow the patient to perform the exercise without interruptions at least 30 times but with a certain degree of effort to complete the activity. The first step will be to determine the exercise or weight that allows them to do the exercise properly about 30 times without stopping yet makes them feel as though they have made an effort by the end.
  - ◦ To be effective, this programme should be followed for 12 weeks. After that time, the tests (the SPPB, GVT and assessment for determining risk of falls) should be repeated.
- Strength and power exercises are shown in Izquierdo et al. [26].

##### Cardiovascular training

- Sets and repetitions:
  - ◦ When the elderly person improves their muscular strength, the cardiovascular exercise programme shall begin.
  - ◦ Walk, alone or with help at usual walking pace, until you are able to walk for 1 min without help.
    - ◦ For example:
      - ▪ Walk 5–10 s, rest 10 s.
      - ▪ Repeat five to seven times.
      - ▪ +
      - ▪ Walk 10–15 s, rest 20 s.
      - ▪ Repeat five to seven times.
    - ◦ Begin by walking for 4 min until you are able to walk for 15 min.
    - ◦ Begin by walking for 8 min until you are able to walk for 30 min.
    - ◦ Walk in sets of 15 min each, resting for 30 s between sets.
- Cardiovascular protocol and exercises in detail according to function are shown in Izquierdo et al. [26].

##### Balance training

- Sets and repetitions:
  - ◦ Remain in the same position and count to 10 (progress up to 30) with each leg. Rest no less than 1 min and no more than 3 min. Repeat with each leg.
  - ◦ +
  - ◦ Walk one set of 10 steps. Stop and rest for 10 s without sitting. Rest no less than 1 min and no more than 3 min. Repeat.
  - ◦ +
  - ◦ Walk in a relaxed way and step over the obstacles. Set up five obstacles to begin. When you finish the walk, begin again. Repeat eight times.
- Intensity and progression:
  - ◦ Change the position of your arms; cross your arms or make a cross shape, for example.
  - ◦ Do the exercises on different surfaces; on a rug, for example.
  - ◦ Close your eyes, but only if someone is near to help you.
- Balance exercises are shown in Izquierdo et al. [26].

##### Flexibility

- Sets and repetitions:
  - ◦ Two sets of three repetitions (remain in the same position for 10 s).
- Intensity and progression:
  - ◦ Stretch until you feel a bit of tension and then remain in the same position for 10–12 s.
  - ◦ Stretch without creating any excessive muscular elongation or articular tension.
  - ◦ Every day.
  - ◦ After the muscular strength and power or cardiovascular exercises.
- Flexibility exercises are shown in Izquierdo et al. [26].

Materials will be also provided to the caregivers and the participants in the Spanish language. All of the materials to be used will be properly adapted to the focused population. This exercise programme has recently launched the Vivifrail App for IOS and Android operating systems, which enables health professionals to assess the functional ability of elderly subjects and help them to prescribe a tailored multi-component physical exercise programme [26, 27] (www.vivifrail.com).

### Outcome measures

#### Primary outcome

The primary outcome measure is the change in functional and cognitive status during the study period. The functional capacity of patients will be evaluated by the Short Physical Performance Battery (SPPB) [34], which evaluates, balance, gait ability and leg strength using a single tool. The total score ranges from 0 (worst) to 12 points (best). The SSPB test has been shown to be a valid instrument for screening frailty and predicting disability, institutionalization and mortality. A total score of less than 10 indicates frailty and a high risk of disability and falls. A 1-point change in the score has clinical relevance [35, 36].

#### Secondary outcomes

Loss of handgrip in the dominant hand is a useful tool for the measurement of functional capacity. This characteristic is a strong predictor of disability, morbidity and mortality as well as one of the components of Fried’s frailty phenotype [4]. Furthermore, the functional status of patients will also be assessed at recruitment with the Barthel Index [37], an international and validated tool of disability. The score ranges from 0 (severe functional dependence) to 100 (functional independence). At baseline, gait ability will be assessed using the 6-m gait velocity test (GVT). Starting and ending limits will be marked on the floor with tapelines for a total distance of 8 m. Participants will be instructed to walk at their self-selected usual pace for two attempts. The best result of both trials will be registered. The first and last metre, considered the warm-up and deceleration phases, respectively, will not be included in calculations of the gait assessment. Dual-task conditions (gait evaluation during the simultaneous performance of a cognitive task) have recently been recognized as a sensitive assessment method for interactions among cognition, gait, falls and frailty. Changes in gait parameters (i.e. gait velocity and gait variability) while performing a dual-task test (dual-task cost) may be early predictors of fall risk [38, 39] and may be useful tools for functional evaluations in frail older patients. Exercise can modify the dual-task cost and, consequently, the fall risk and functional capacity [40]. The dual-task paradigm [37] will be used in the 6-m habitual GVT. Two trials will be conducted to assess gait velocity while the patient is performing a verbal or counting task (verbal GVT and arithmetic GVT, respectively). During the verbal dual-task condition (verbal GVT), we will measure gait velocity while participants are naming animals aloud. During the arithmetic dual-task condition (arithmetic GVT), we will assess gait velocity while participants are counting backwards aloud from 100 in ones. The cognitive score will be measured by counting the number of animals named (dual-task with verbal performance) or determining how many numbers were counted backwards (dual-task with arithmetic performance). Isometric upper limb (dominant hand grip) and lower limb (right knee extensors and hip flexors) muscle strength will be measured using a manual dynamometer. Maximal dynamic strength will be assessed using the 1RM test in the bilateral leg press exercise using exercise machines (Matrix, Johnson Health Tech, Ibérica, S.L., Torrejón de Ardoz, Spain; and Exercycle S.L., BH Group, Vitoria, Spain). In the first assessment, the subjects will warm up with specific movements for the exercise test. Each subject’s maximal load will be determined in no more than five attempts, with a 3-min recovery period between attempts. After the 1RM values are determined, the subjects will perform 10 repetitions at maximal velocity and at intensities of 50% of 1RM to determine the maximum power (W) and the loss of power during the 10 repetitions in the leg press exercise [41]. The power will be recorded by connecting a velocity transducer to the weight plates (T-Force System, Ergotech, Murcia, Spain). During all neuromuscular performance tests, strong verbal encouragement will be given to each subject to motivate them to perform each test action as optimally and rapidly as possible. Distribution of the training sessions throughout the day should minimize cumulative fatigue and help to maintain adherence. Adherence to the exercise programme will be documented in an individual daily log of the sessions. Changes in cognitive-affective status after the intervention will be measured using the Spanish validated versions of the Minimental Cognitive Exam (MEC-Lobo) [42] in the case of dementia and the MOCA [43] in the case of MCI. The Yesavage GDS [44] and Trail Making part A [45] will be performed to assess depression and executive dysfunction. During functional tasks (such as balance, gait and rising from a chair) and cognitive evaluations (dual tasking), an inertial sensor unit (IU) will be attached over the lumbar spine (L3) to record the acceleration data in control and intervention participants [46, 47]. Primary and secondary variables are presented in Table 1.

## Discussion

Frailty and cognitive impairment are two very common geriatric syndromes in elderly patients and are frequently related and overlapped. Functional decline and disability are major adverse outcomes of these conditions. Exercise is a potential intervention for both syndromes. If our hypothesis is correct, the relevance of this project is that the results can contribute to understanding that the individualized multicomponent exercise programme (VIVIFRAIL) for frail elderly patients with cognitive impairment is more effective in reducing functional and cognitive impairment than conventional care. Moreover, our study may be able to show that an innovative individualized multicomponent exercise prescription for these high-risk populations is plausible, having at least similar therapeutic effects to other pharmacological and medical prescriptions.

Traditionally, health systems and policies focus attention on the management of chronic conditions as the central paradigm of care for the elderly. On the other hand, evidence coming from a geriatrics perspective in the last 30 years and international health organizations such as the World Health Organization (WHO) has emphasized the significance of maintaining functional capacity, recently renamed as intrinsic capacity, as the central objective and focus of all health systems and policies for elderly populations. Thus, this trial is in line with these objectives and recommendations. The performance of exercise programmes can improve quality of life and maintain independence in activities of daily living, and can potentially improve cognitive function and prevent adverse health outcomes (i.e. falls and fractures, hospitalizations and nursing homes admissions) in this high-risk population. In addition, the clinical impact of this trial can be significant if we help to modify and shift the traditional management of this population from an illness model to a more person-centred and functionally oriented perspective. Moreover, if our hypotheses are correct, the prescription of individualized exercise can be routinely included in the clinical practice of health professionals tending to elderly frail patients with cognitive complaints.

An important aspect of our trial is the inclusion of elderly patients with mild cognitive decline and dementia. So far, the majority of trials in aged frail participants with these conditions have been routinely excluded. The inclusion of participants with cognitive impairment in addition to frailty makes the trial novel and unique with notable external validity compared with other previous trials in assessing the effect of individualized exercise programmes on functional capacity, activities of daily living and cognitive function.

Another remarkable characteristic of our study is the utilization of an interdisciplinary team (geriatricians, nurses, physiotherapy, engineers) that manages not only the clinical aspects but also the physiotherapy and engineering kinematics. This aspect enables the translation and application of our results to different clinical practices and enables the establishment of new protocols related to the ageing process for different health professionals and for monitoring functional changes objectively.

To date, randomized clinical trials with exercise interventions in aged participants have been heterogeneous (sometimes with confusing details related to the exercise programme). An innovative aspect of our trial is the performance of a detailed and well-designed exercise intervention using the VIVIFRAIL methodology that can be easily extrapolated to other clinical settings and scenarios like community centres, day care centres, nursing homes and hospitals. If our results confirm our hypothesis, this randomized clinical trial could help to disseminate the “VIVIFRAIL exercise recipe” worldwide to frail seniors with cognitive decline.

### Dissemination

We will disseminate the results of our study via presentations at international conferences and articles in peer-reviewed journals. The study will be implemented and reported in accordance with the Standard Protocol Items: Recommendations for Interventional Trials (SPIRIT) guidelines.

### Future directions

In the near future, this project offers the opportunity to test and disseminate “in real life” a novel prescription exercise tool (VIVIFRAIL) in the very vulnerable population of frail elders with cognitive decline. The project will yield direct information about the effects of a specific and individualized designed exercise programme in those geriatric conditions. In the long term, we expect this trial can help to shift actual medical care from the traditional disease perspective to a more functional management. Another important expected result in our project is to generalize the prescription of exercise in this population in order to prevent disability. Finally, with the actual target of a novel era of precision medicine, our results could be a significant step forwards to move towards a “precise prescription of exercise recipe” for older patients with frailty and cognitive decline impairment.

### Trial status

The trial commenced recruitment in September 2017 and is currently open for recruitment. Recruitment will cease when 240 participants have been randomized. It is anticipated that this target will be reached by September 2019.

## Additional file

Additional file 1: SPIRIT 2013 Checklist: Recommended items to address in a clinical trial protocol and related documents (PDF 68 kb)

## Data Availability

The datasets generated during and/or analysed during the current study are available from the corresponding author on reasonable request.
